# Epidemiology of dental caries in permanent dentition: evidence from a population-based survey in Egypt

**DOI:** 10.1186/s12889-022-14844-9

**Published:** 2022-12-27

**Authors:** Mona Ahmed Abdel Fattah, Muhammad Helmi Barghouth, Mariem Osama Wassel, Omar Hassan Deraz, Ahmed Essam Khalil, Hazem Magdy Sarsik, Ahmed Mohamed Ali Mohsen, Amr Shaaban Qenawy, Reham Khaled Abou El Fadl

**Affiliations:** 1 Ministry of Health and Population, 3 Magles El Shaab Street, Cairo, Egypt; 2 Faculty of Dentistry, Ain Shams University, Organization of African Unity st. Abbasia, 11566 Cairo, Egypt; 3 Integrative Epidemiology of Cardiovascular Disease, Université de Paris, INSERM U970, 56 Rue Leblanc, 75015 Paris, France; 4 British University in Egypt, Suez Desert Road El Sherouk City, Cairo, 11837 Egypt; 5 Faculty of Dentistry, Tanta University, Tanta Qism 2, Tanta, Gharbia Governorate, Cairo, 6624033 Egypt

**Keywords:** Caries, Permanent teeth, Epidemiology, DMFT, Egyptians, Upstream health determinants

## Abstract

**Background:**

In recognition of the risk factors common between oral diseases and various chronic conditions and the intersection between oral health and some sustainable development goals, the current cross-sectional study was designed to quantify the burden of dental caries and identify factors associated with its occurrence in permanent teeth.

**Methods:**

Using data from Egypt's population-based survey (2013–2014), two individual-level outcomes; past caries experience (DMFT > 0) and presence of untreated carious lesions (DT > 0) were assessed using the WHO basic methods for oral health surveys. Information on potential explanatory variables including sociodemographic characteristics, exposure to fluoridated water, dental attendance, and dental anxiety was gathered using a structured questionnaire. Stratified multistage cluster random sampling was used to recruit survey participants. Multivariable logistic regression was performed to identify significant potential risk factors for caries in the permanent dentition of Egyptians.

**Findings:**

A total of 9,457 participants were included of which 70.3% had at least one untreated carious lesion. After adjusting for all covariates, analphabetic Egyptians were found to have significantly higher odds of caries experience in permanent dentition DMFT > 0 (OR 1.54, 95% CI [1.20–1.98]), DT > 0 (OR 1.62, 95% CI [1.32–2.00]). Males, however, had significantly lower caries risk DMFT > 0 (OR 0.75, 95% CI [0.67–0.85]), DT > 0 (OR 0.81, 95% CI [0.73–0.89]) when compared to females. Regarding age, mean DMFT scores were significantly lower in age groups (6–15 years) (OR 0.03, 95% CI [0.014; 0.082]), (16– 20 years) (OR 0.09, 95% CI [0.037; 0.23]), and (21–35 years) (OR 0.22, 95% CI [0.09; 0.53]) than among people ≥ 60 years.

**Conclusion:**

Addressing individual-level caries risk factors should be complemented by addressing upstream factors to reduce burden of untreated dental caries among Egyptians.

## Introduction

Over the last two decades, oral disorders have undergone significant epidemiologic shifts [1] and were deemed endemic affecting 3.6 billion people globally [2]. Though largely preventable, dental caries remains a discernible health challenge worldwide. According to the Global Burden of Disease study 2019, untreated dental caries in permanent dentition was the most prevalent health problem, affecting around 2 billion people with over 45% increase in the number of cases from 1990 to 2019 [3]. Furthermore, while the global economic burden of dental disorders collectively exceeded $540 billion in 2015, untreated caries in permanent teeth accounted solely for 11% of global productivity losses attributed to those disorders [4].

Investigating both contextual and behavioral determinants of health conditions at a population level and understanding the level of their contribution to disease burden is imperative for devising future primary prevention strategies and addressing health inequalities. Recently, in recognition of the multifaceted burden of oral diseases worldwide, the urgency of understanding social determinants of poor oral health was underscored in the 74^th^ World Health Assembly in 2021 to redirect attention to the root causes of oral health disparities [5]. Despite the evidence that not all determinants of a health-disease process can be disclosed at an individual level, [6] to date, oral health research has focused primarily on exploring lifestyle factors such as dietary and oral hygiene practices involved in escalating the global burden of dental caries [7, 8] while putting less emphasis on social structures that shape those risk factors.

In most countries, conducting population-based surveys or implementing surveillance systems to monitor oral health status are not considered a public health priority [1]. In Egypt, until 2013, epidemiologic data about the nationwide prevalence of oral diseases was insufficient and scattered, thus thorough investigations were needed to estimate the effects of multiple determinants on their incidence.

The current study had a two-fold objective: i) estimating the prevalence of dental caries in permanent dentition of Egyptians, and ii) investigating potential oral health determinants that contribute to the increased risk of caries experience. We hypothesize that specific contextual and socioeconomic factors would influence caries experience in permanent dentition among Egyptians.

## Methods

### Study design

The present cross-sectional study is nested in a national oral health survey conducted in Egypt between September 2013 and May 2014 with the support of the Central Administration of Dentistry, Ministry of Health and Population (MOHP), and the World Health Organization (WHO), country office [9]. The study was carried out according to the principles of the Declaration of Helsinki on experimentation pertaining to human subjects (version 2013) and reported according to the Strengthening the Reporting of Observational Studies in Epidemiology (STROBE) guidelines.

### Study sample

For the population-based survey, the sample size was calculated according to Egypt's Census dataset of 2013, provided by the Egyptian Central Agency for Public Mobilization and Statistics (CAPMAS). Accordingly, 10,144 participants—of which 53% resided in urban areas—were included in the final survey to yield a 95% confidence level, a sample power of 99.32%, and a marginal error of 0.68%.

The survey design included a stratified multistage cluster random sampling approach. Sampling took place in three stages based on the Egyptian administrative divisions. Egypt is geographically divided into four regions; Lower Egypt, Upper Egypt, Desert/Frontier and Civilized regions comprising a total of 27 governorates. Each governorate is further divided into rural and urban areas except for five governorates which include only urban areas. One governorate “North Sinai” was excluded from sample collection due to security reasons. The sampling frame from which the primary sampling units were randomly selected for the survey was obtained from CAPMAS and consisted of a list of all localities in each governorate. The second stage of sampling comprised 160 gathering points including healthcare facilities as well as a few households and workplaces. For the final sampling stage, systematic random sampling was used to select potential participants from individuals present in each gathering point on the day of recruitment. During data collection, edentulous individuals and those seeking dental care—to avoid selection bias—were excluded from the survey. As the current analysis addresses only caries experience in permanent teeth, children in the primary dentition stage were also excluded from the study sample, yielding 9,457 participants.

### Study variables

To test study hypothesis, we investigated the effect of potential determinants on the participants' caries experience using two individual-level dental outcomes: a) past caries experience indicated by the cumulative Decayed, Missing, and Filled Teeth (DMFT) index score; b) untreated caries lesions indicated by the number of decayed teeth (DT component of the index).

Face-to-face interviews were conducted to collect data on sociodemographic characteristics such as age, gender, and place of residence (rural/urban) using a structured questionnaire in simple Arabic language. In the case of minors, the questionnaire was answered by guardians instead. Educational attainment; a combined variable (hereinafter referred to as educational level) comprising the educational level of adults and the paternal educational level of minors was used as an indicator of socioeconomic position (SEP), and included the following categories: *analphabetic, high school or less, and two-year academy, college or above.* As most Egyptian households are male headed (83%) [10] and given the inverse relationship between a household's poverty risk and paternal educational level, the latter was used to denote the SEP of minors [11].

Source of drinking water, dental anxiety, and frequency of dental visits were examined as further explanatory variables. Sources of drinking water included *filtered* and *unfiltered tap water*, as well as *bottled*, *well and mixed-source water*. The frequency of dental visits was determined based on whether participants' last dental visit was within *6 months*, *6–12 months*, *12–24 months*, or *more than 24 months* before participating in the survey or if they have *never visited a dentist*. Dental anxiety was tested using a single question; “Do you experience any anxiety when going to the dentist?” which was asked to the participants themselves or to guardians in case the participants were minors.

To assess the oral health status of participants, clinical examination was done as per the WHO basic methods for oral health surveys [12]. Visual and tactile examination of the crowns and roots of erupted permanent teeth was performed, excluding third molars. Plane surface mouth mirrors and blunt ball-ended WHO CPI probes were used to remove debris from tooth fissures and assess the presence of fissure sealants or defective restorations without jeopardizing the surface integrity of the undermined enamel overlying early carious lesions.

Teeth were given a score ranging from 0 to 9 only when their incisal edges or occlusal surfaces were completely visible. Sound non-carious teeth were assigned score 0. Teeth showing signs of caries such as enamel breakdown, a distinct cavity, undermined enamel, or an underlying dentin shadow were assigned score 1. Restored teeth were scored according to restoration integrity where restored non-carious teeth were assigned score 2 whereas carious ones were assigned score 3. Missing teeth were scored according to reasons for extraction reported by the participants; teeth missing due to caries were assigned score 4 whereas those missing due to other reasons were given score 5. Sealed non-carious teeth were assigned score 6 while abutment teeth without evidence of caries were given score 7. Unerupted permanent teeth and teeth that could not be examined were given scores 8 and 9 respectively [12]. Assigned scores were then converted using DMFT index scoring where teeth given scores 1 and 3 were assigned to the "Decayed" category, whereas those with scores 2 and 4 were assigned to the "Filled" and the "Missed" categories respectively.

Data collection was carried out by 20 investigators recruited from the MOHP and the Faculty of Dentistry, Ain Shams University, Cairo, Egypt. All investigators partook in a five-day training session on the examination procedures. Multiple calibrations were performed during data collection where investigators examined the same sample of individuals and variability among examiners was assessed. Inter-examiner reliability was deemed satisfactory at kappa values of 0.75 or greater [13].

### Statistical analysis

Unweighted data analysis was performed using IBM1 SPSS1 (SPSS Inc., IBM Corporation, NY, USA) Statistics Version 19 for Windows with a significance level of *P* < 0.05. Descriptive statistics were performed to determine caries prevalence in different population groups. Mean scores, standard deviations (SD) and 95% confidence intervals (95% CI) were reported for the overall DMFT index score as well as for all index categories separately across the entire study sample and unpaired t-test or one way ANOVA test were used to compare between mean values.

Multivariable logistic regression models were created for past caries experience (all DMFT index components) and untreated disease (DT index component) for which the odds ratios (OR) and 95% CI were estimated as measures of association. Both models were adjusted for well-known caries risk factors including frequency of sugar consumption, tooth brushing, and smoking as well as other covariates such as age, gender, educational level, place of residence, source of drinking water, dental anxiety, and frequency of dental visits.

## Results

### Participants' characteristics and background information

Descriptive statistics of the study sample are shown in (Table 1). The sample included 9,457 participants of which 1,731 participants had both primary & permanent teeth including 52 five-year-old participants who had at least one fully erupted permanent tooth. The mean sample age was 28.8 ± 16.0 (range 5–85), 50.90% were females and 46.60% resided in rural settings. Dental anxiety was reported by 46.52% of the participants and 39% stated visiting the dentist recently (i.e., within the past six months). 78.20% of the study sample had DMFT > 0 with total mean value 5.5 ± 5.7, 70.30% had at least one untreated carious permanent tooth (DT > 0) with mean value 3.0 ± 3.4.

**Table 1:** Prevalence of dental caries stratified by sociodemographic characteristics and other relevant oral health indicators (*N* = 9,457)

		**Caries experience**					
		**N** **(%)**	**DMFT > 0% Prevalence**	**D mean score, SD** **(95% CI)**	**M mean score, SD** **(95% CI)**	**F mean score, SD** **(95% CI)**	**DMF mean score, SD** **(95% CI)**
**Geographic region**	Lower Egypt	4083 (43%)	3198 (78.3%)	2.8 (3.2) (2.7–2.9)	1.9 (3.5) (1.8–2)	0.6 (1.6) (0.6–0.7)	5.3 (5.5) (5.2–5.5)
	Upper Egypt	3392 (35.86%)	2693 (79.4%)	3.4 (3.7) (3.3–3.6)	2 (3.7) (1.9–2.2)	0.4 (1.2) (0.38–0.42)	5.9 (6) (5.7–6.1)
	Civilized	1805 (19%)	1351 (74.8%)	2.6 (3.2) (2.4–2.7)	1.7 (3.4) (1.6–1.9)	0.8 (1.7) (0.7–0.8)	5.1 (5.5) (4.8–5.3)
	Desert/ Frontier	177 (1.87%)	154 (87%)	3.8 (3.7) (3.2–4.3)	1.4 (3) (1–1.9)	0.6 (2.1) (0.3–1)	5.9 (5.3) (5.1–6.6)
	***p*-value **^**b**^		** < 0.001**	** < 0.001**	**0.01**	** < 0.001**	** < 0.001**
**Age**	2–5 yrs.^a^	52 (0.55%)	13 (25%)	0.6 (1.1) (0.2–0.9)	-	-	0.6 (1.1) (0.2–0.9)
	6–15 yrs	2862 (30.26%)	1520 (53.1%)	1.6 (2.1) (1.5–1.6)	0.1 (0.3) (0–0.1)	0.1 (0.6) (0.09–0.11)	1.7 (2.3) (1.6–1.8)
	16–20 yrs	694 (7.33%)	509 (73.3%)	3.1 (3.3) (2.9–3.4)	0.2 (0.5) (0.1–0.2)	0.3 (1.3) (0.2–0.4)	3.6 (3.7) (3.3–3.9)
	21–35 yrs	2614 (27.64%)	2293 (87.7%)	4 (3.9) (3.9–4.2)	1.2 (2) (1.1–1.3)	0.8 (1.7) (0.7–0.8)	6 (5) (5.8–6.2)
	36–45 yrs	1427 (15.08%)	1335 (93.6%)	3.7 (3.5) (3.5–3.9)	3.1 (3.5) (2.9–3.3)	1 (1.9) (0.9–1.1)	7.8 (5.6) (7.5–8.1)
	46–59 yrs	1601 (16.92%)	1525 (95.3%)	3.3 (3.4) (3.1–3.5)	5.1 (4.8) (4.9–5.3)	0.9 (1.8) (0.8–1)	9.3 (6.3) (9–9.6)
	≥ 60 yrs	207 (2.18%)	201 (97.1%)	3.7 (3.8) (3.2–4.2)	9.2 (6.9) (8.3–10.2)	0.5 (1.8) (0.2–0.7)	13.4 (8) (12.3–14.5)
	***p*-value**		** < 0.001**	** < 0.001**	** < 0.001**	** < 0.001**	** < 0.001**
**Gender**	Male	4640 (49.06%)	3508 (75.6%)	2.7 (3.3) (2.6–2.8)	1.9 (3.6) (1.8–2)	0.5 (1.4) (0.4–0.5)	5.1 (5.7) (4.9–5.2)
	Female	4817 (50.93%)	3888 (80.7%)	3.3 (3.5) (3.2–3.4)	1.9 (3.4) (1.8–2)	0.7 (1.6) (0.6–0.7)	5.9 (5.6) (5.7–6)
	***p*-value**		** < 0.001**	** < 0.001**	0.830	** < 0.001**	** < 0.001**
**Place of residence**	Rural	4413 (46.66%)	3465 (78.5%)	3.2 (3.6) (3.1–3.3)	2 (3.6) (1.9–2.1)	0.4 (1.2) (0.3–0.4)	5.6 (5.8) (5.4–5.7)
	Urban	4978 (52.63%)	3878 (77.9%)	2.8 (3.2) (2.7–2.9)	1.8 (3.4) (1.8–1.9)	0.7 (1.8) (0.7–0.8)	5.4 (5.5) (5.3–5.6)
	***p*-value**		0.47	** < 0.001**	0.12	** < 0.001**	0.20
**Educational** **level**	Analphabetic	782 (8.26%)	653 (83.5%)	4 (4) (3.7–4.3)	3.1 (4.7) (2.8–3.4)	0.2 (1) (0.1–0.3)	7.3 (6.7) (6.8–7.7)
	High school or less	5423 (57.34%)	4320 (79.7%)	3.2 (3.5) (3.1–3.3)	2.1 (3.8) (2–2.2)	0.5 (1.3) (0.4–0.5)	5.8 (5.8) (5.6–5.9)
	Two years academy, college or above	2888 (30.53%)	2206 (76.4%)	2.6 (3.1) (2.4–2.7)	1.3 (2.6) (1.2–1.4)	0.9 (2) (0.9–1)	4.8 (5) (4.6–5)
	***p*-value**		** < 0.001**	** < 0.001**	** < 0.001**	** < 0.001**	** < 0.001**
**Frequency of dental visits**	Within the past 6 months	3707 (39.19%)	3069 (82.8%)	3.3 (3.5) (3.2–3.4)	2.5 (4) (2.3–2.6)	0.9 (1.9) (0.8–0.9)	6.6 (6.1) (6.4–6.8)
	6 to 12 mos	1181 (12.48%)	992 (84%)	3.3 (3.4) (3.1–3.5)	2.1 (3.4) (1.9–2.3)	0.8 (1.8) (0.7–0.9)	6.1 (5.6) (5.8–6.5)
	1–2 yrs. ago	986 (10.42%)	817 (82.9%)	3.1 (3.4) (2.9–3.3)	2.2 (3.7) (2–2.4)	0.6 (1.5) (0.5–0.7)	5.9 (5.6) (5.6–6.3)
	More than 2 yrs	1974 (20.87%)	1585 (80.3%)	2.9 (3.3) (2.8–3.1)	1.8 (3.5) (1.7–2)	0.3 (1) (0.3–0.4)	5.1 (5.4) (4.9–5.4)
	Never	1483 (15.68%)	825 (55.6%)	2.1 (3) (1.9–2.2)	0.2 (1.1) (0.1–0.2)	0 (0.2) (0–0)	2.3 (3.3) (2.1–2.4)
	***p*-value**		** < 0.001**	** < 0.001**	** < 0.001**	** < 0.001**	** < 0.001**
**Source of drinking water**	Filtered tap water	1434 (15.16%)	1091 (76.1%)	2.6 (3.1) (2.4–2.8)	1.6 (3.3) (1.4–1.8)	1 (2.1) (0.9–1.1)	5.2 (5.5) (4.9–5.5)
	Unfiltered tap water	7047 (74.51%)	5548 (78.7%)	3.1 (3.5) (3–3.2)	2 (3.6) (1.9–2.1)	0.5 (1.3) (0.47–0.53)	5.6 (5.7) (5.5–5.8)
	Bottled water	136 (1.43%)	110 (80.9%)	3 (3.6) (2.4–3.6)	1.2 (2.6) (0.7–1.6)	1.1 (1.9) (0.7–1.4)	5.2 (5.4) (4.3–6.2)
	Well water	452 (4.77%)	351 (77.7%)	2.9 (3.4) (2.6–3.2)	1.7 (3.6) (1.3–2)	0.3 (1.1) (0.2–0.4)	4.8 (5.5) (4.3–5.4)
	Mixed water	349 (3.69%)	265 (75.9%)	2.6 (3.1) (2.3–3)	1.1(2.4) (0.9–1.4)	0.9 (2.2) (0.7–1.1)	4.7 (4.9) (4.2–5.2)
	***p*-value**		0.16	** < 0.001**	** < 0.001**	** < 0.001**	** < 0.001**
**Dental Anxiety**	Yes	4400 (46.52%)	3494 (79.4%)	3.2 (3.5) (3.1–3.3)	1.8 (3.4) (1.7–2)	0.6 (1.5) (0.5–0.6)	5.7 (5.7) (5.5–5.8)
	No	4658 (49.25%)	3661 (78.6%)	2.8 (3.3) (2.7–2.9)	2 (3.7) (1.9–2.1)	0.6 (1.6) (0.6–0.7)	5.5 (5.7) (5.3–5.6)
	Unsure	254 (2.68%)	131 (51.6%)	1.9 (2.9) (1.6–2.3)	0.3 (1.4) (0.1–0.5)	0.1 (0.4) (0–0.1)	2.3 (3.5) (1.9–2.7)
	***p*-value**		** < 0.001**	** < 0.001**	** < 0.001**	** < 0.001**	** < 0.001**

^a^ All subjects of age range 2 to 5 years who had permanent teeth were 5 years old

^b^ANOVA was used to test significance of differences in mean values for DMF and Chi-square test was used to measure significance of differences in prevalence of past caries experience (DMF > 0)

The prevalence and mean scores of past caries experience (DMFT > 0) and mean scores for untreated caries (DT > 0), filled and missing teeth due to caries are also provided in Table 1 stratified by age, gender, geographic location, place of residence, educational level, frequency of dental visits, source of drinking water and dental anxiety.

### Associations with past caries experience and untreated carious lesions

**Fig. 1:**
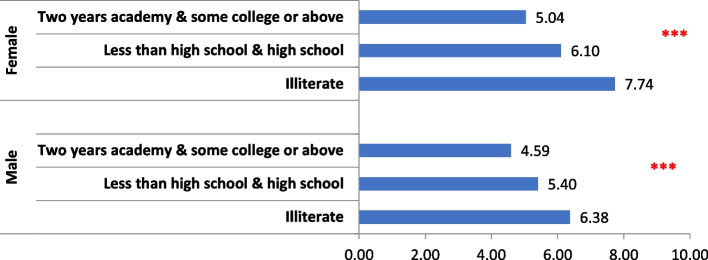
Bar chart showing the relation between educational level and mean DMFT stratified by gender (*** p-value ≤ *0.00)*

**Fig. 2:**
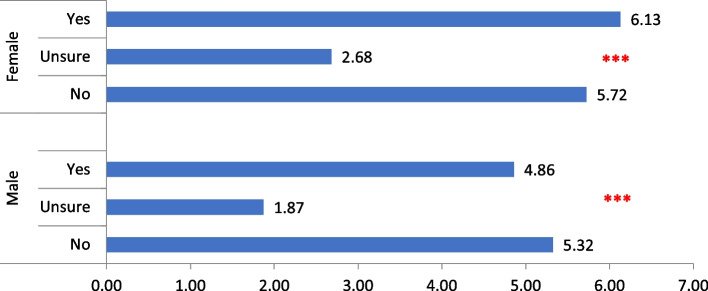
Bar chart showing the relation between dental anxiety and mean DMFT stratified by gender (*** p-value ≤ *0.00)*

A significant negative association was detected between mean DMFT scores and level of education for both genders (Fig. 1). In Fig. 2, while mean DMF scores were significantly higher among dentally anxious female participants, dental anxiety was negatively associated with caries experience in males.

Tables 2 and 3 show the results from the multivariable logistic regression analysis for past and present caries experience respectively. The likelihood of having a DMFT score > 0 was lower in young age groups; (6–15 yrs.) (OR 0.03, 95% CI [0.01; 0.08]), (16–20 yrs.) (OR 0.09, 95% CI [0.04; 0.23]), (21–35 yrs.) (OR 0.22, 95% CI [0.09; 0.53]) than among participants ≥ 60 years. On the other hand, the differences in odds of having untreated caries (DT > 0) were non-significant across age groups (16–20) years (OR 0.70 95% CI [0.47; 1.05]), (21–35) years (OR 1.09 95% CI [0.75; 1.58]), (36–45) years (OR 1.18 95% CI [0.80; 1.74], or (46–59) years (OR 0.92, 95% CI [0.63; 1.35]) when compared to participants ≥ 60 years.

**Table 2:** Multivariable (adjusted) Logistic Regression analysis for association between past caries experience in permanent dentition (DMF > 0) and potential risk factors

***DMF* > *0***		*Std. Error*	*Sig.*	* OR*	*95% CI for OR *	
					*Lower Bound*	*Upper Bound*
**Geographic region**	Lower Egypt	0.27	**0.01**	0.47	0.28	0.80
	Upper Egypt	0.27	0.05	0.58	0.34	1.00
	Civilized	0.27	**0.002**	0.42	0.25	0.72
**Age**	2–5 Years	0.58	** < 0.001**	0.01	0.003	0.03
	6–15 Years	0.46	** < 0.001**	0.03	0.014	0.082
	16–20 Years	0.47	** < 0.001**	0.09	0.037	0.23
	21–35 Years	0.46	**0.001**	0.22	0.09	0.53
	36–45 Years	0.47	0.05	0.40	0.16	1.01
	46–59 Years	0.47	0.18	0.53	0.21	1.33
**Gender**	Male	0.06	** < 0.001**	0.75	0.67	0.85
**Place of residence**	Rural	0.07	0.60	0.96	0.84	1.11
**Educational level**	Analphabetic	0.13	**0.001**	1.54	1.20	1.98
	Up to high School	0.07	0.06	1.14	1.00	1.30
**Frequency of dental visits**	Never visited	0.08	** < 0.001**	0.41	0.35	0.48
	More than 2 years	0.08	0.20	0.90	0.76	1.06
	1–2 Years ago	0.11	**0.02**	1.30	1.05	1.62
	6 months to 1 years	0.11	0.05	1.23	1.00	1.51
**Source of drinking water**	Mixed Water	0.15	0.47	0.90	0.67	1.21
	Bottled Water	0.26	0.14	1.45	0.88	2.39
	Well Water	0.14	**0.01**	1.43	1.08	1.88
	Filtered Tap Water	0.09	0.79	1.02	0.86	1.21
**Dental Anxiety**	Yes	0.06	** < 0.001**	1.29	1.14	1.46

N.B. Reference category of geographic region is Desert/Frontier, Reference category of age groups is ≥ 60 years, Reference category of educational level is College or above, Reference category of frequency of dental visits is within the past 6 months, Reference category of source of drinking water is non-filtered tap water

**Table 3:** Multivariable (adjusted) Logistic Regression analysis for association between untreated caries in permanent dentition (D > 0) and potential risk factors

***D* > *0***		*Std. Error*	*Sig.*	*OR*	*95% CI for OR *	
					*Lower Bound*	*Upper Bound*
**Geographic** **region**	Lower Egypt	0.23	**0.001**	0.48	0.31	0.76
	Upper Egypt	0.23	**0.03**	0.60	0.39	0.95
	Civilized	0.23	** < 0.001**	0.42	0.27	0.66
**Age**	2–5 Years	0.40	** < 0.001**	0.09	0.04	0.19
	6–15 Years	0.19	** < 0.001**	0.28	0.20	0.41
	16–20 Years	0.21	0.09	0.70	0.47	1.05
	21–35 Years	0.19	0.67	1.09	0.75	1.58
	36–45 Years	0.20	0.40	1.18	0.80	1.74
	46–59 Years	0.19	0.66	0.92	0.63	1.35
**Gender**	Male	0.05	** < 0.001**	0.81	0.73	0.89
**Place of residence**	Rural	0.06	0.72	0.98	0.87	1.10
**Educational** **Level**	Analphabetic	0.11	** < 0.001**	1.62	1.32	2.00
	Up to high School	0.06	**0.001**	1.22	1.09	1.36
**Frequency of Dental visits**	Never visited	0.07	** < 0.001**	0.53	0.46	0.61
	More than 2 years	0.07	0.26	0.93	0.81	1.06
	1–2 Years ago	0.09	0.32	1.09	0.92	1.30
	6 months to 1 years	0.08	0.21	1.11	0.94	1.31
**Source of drinking water**	Mixed-source Water	0.13	0.27	0.87	0.67	1.12
	Bottled Water	0.21	0.53	1.14	0.76	1.73
	Well Water	0.12	0.23	1.15	0.91	1.46
	Filtered Tap Water	0.07	0.35	0.94	0.81	1.08
**Dental anxiety**	Yes	0.05	** < 0.001**	1.32	1.19	1.46

N.B. Reference categories of independent variables are similar to Table 2

Prevalence odds ratios showed that analphabets had significantly higher DMFT > 0 and DT > 0 than people with a college education and above; (OR 1.54, 95% CI [1.20; 1.98]) and DT >0 (OR 1.62, 95% CI [1.32; 2.00] respectively in the multivariable logistic regression analysis. Dental anxiety was also positively associated with caries experience where dentally anxious participants had significantly higher odds of worse past and present caries experience; (OR 1.29, 95% CI [1.14; 1.46]) and (OR 1.32, 95% CI [1.19; 1.46]) respectively. Besides, gender differences were significant, with lower odds (OR 0.75, 95% CI [0.67; 0.85]) and (OR 0.81, 95% CI [0.73; 0.90] for DMFT > 0 and DT > 0 respectively among males.

## Discussion

Findings from Egypt’s national oral health survey 2013–2014, provide the first representative information on caries experience in permanent dentition in the Egyptian population since the early nineties. Our findings suggest that, the burden of dental caries in permanent teeth (DMFT score 5.5 ± 5.7) is too high, for example, when compared to the overall mean DMFT reported by a meta-analysis on the caries experience in a number of countries in East Africa that reached 1.9 [14]. Moreover, decayed teeth (D) constituted the largest fraction of the DMFT scores in different groups of Egyptian population when stratified by age, gender, educational level, and place of residence.

Globally, it has been reported that the prevalence of untreated caries in permanent dentition peaked at age 20 to 24 years [15] which is in accordance with the findings of our study. One plausible explanation could be that seeking dental care by Egyptians is mainly symptom-driven, hence individuals tend to postpone dental visits until they experience pain. Since caries is a slowly progressing disease, lesions in recently erupted permanent teeth could take years before reaching advanced stages which are painful [16]. This is further supported by the subsequent declining prevalence of untreated carious lesions among those over the age of 24 years due to increased dental care utilization. Furthermore, because DMFT index exclusively considers carious teeth and not those at risk of developing caries, younger age groups are expected to have lower DT scores as they are more likely to have fewer erupted permanent teeth which have not been exposed long enough to caries-promoting factors such as cariogenic foods [17].

Our results also confirm the study hypothesis by revealing that SEP, dental anxiety, gender and access to fluoridated water have significant influence on caries experience in the permanent dentition among Egyptians. Adjusted regression analysis showed that low educational attainment significantly increased the odds of having worse past caries experience and more untreated carious lesions. Based on estimates of burden of dental caries from the global burden of disease study 2019, the prevalence of caries in permanent dentition was negatively associated with the sociodemographic index quintile [3]. Dental caries is a "deprivation disease" disproportionately affecting the disadvantaged lacking access to oral care [18] Empirical evidence indicates that SEP as determined by educational attainment, income, or occupation is inversely associated with caries experience in different age groups [18, 19]. Findings from a community-based survey revealed that, among Ethiopian adults, risk of caries experience decreases with higher level of education [20].

In Egypt, due to the steady increase in poverty rates reaching 27.80% as of mid-2016, [21] the government had been subsidizing basic commodities to enable low-income households to fulfill their daily nutritional requirements. This in turn has led to overconsumption of calorie-rich food items particularly oil, sugar and bread causing the beneficiaries' diet to be increasingly unbalanced and cariogenic [22].

Another mechanism underlying the association between caries and SEP could be found in the concept of 'health literacy' which comprises a set of skills that enables people to make informed decisions to maintain and improve their health and well-being [23]. Educational attainment is known to capture people's levels of knowledge and skills [24] and their abilities to adhere to either negative or positive health behaviors and treatment protocols [25]. Oral health illiteracy was found to be correlated with low socioeconomic level and individuals with limited oral health knowledge were less likely to comprehend oral self-care behaviors and tended to forgo seeking routine dental care placing themselves at high risk for oral diseases [26].

Dental anxiety is also known to be strongly associated with avoidance behaviors towards dental care and subsequently higher risk of experiencing oral problems including dental caries which is in line with our findings [27]. By definition, dental anxiety is a phenomenon which affects all ages and includes a multitude of causes such as exposure to past medical or dental trauma or aversive life events such as abuse or psychological distress due to negative social or environmental events [28–30].

Noticeably, 46.5% of our sample reported being dentally anxious and the likelihood that they are mostly females, at a young age or of low SEP is a common finding in literature on dental anxiety [31]. This could partially explain the relationship observed in our study between female gender and caries incidence (Fig. 2). As gender comprises culture-bound beliefs, roles and behaviors, those factors might have further reinforced this relationship. In a patriarchal society as Egypt, it is not uncommon that women's access to healthcare, particularly those who are socially disadvantaged, would be restricted owing to lack of decision-making power, limited mobility, and time constraints due to intense unpaid care and domestic work.

**Fig. 3:**
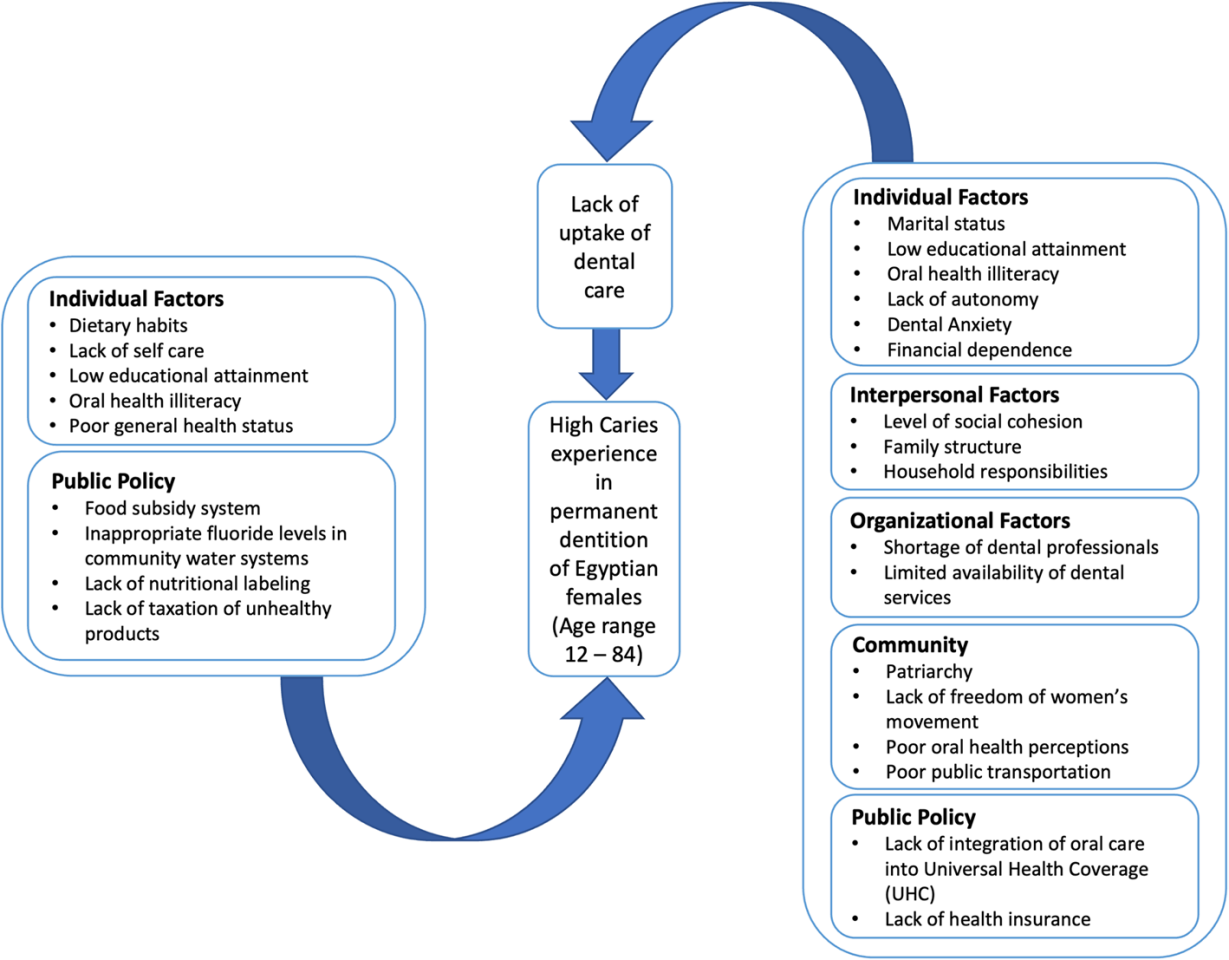
Conceptual framework of direct and indirect factors affecting caries experience in Egyptian females: Socioecological Model

Moreover, in Egypt, costs of most dental services are relatively high and as they are mostly funded through out-of-pocket payments, households are more likely to incur catastrophic dental health expenditures and impoverishment [32]. Hence, financial constraints might have played a pivotal role when social class intersects with gender preventing economically dependent women from poor households from accessing dental care which could have subsequently led to the increased unmet oral care needs among this vulnerable group (Fig. 3). This, also, underscores the importance of prevention in vulnerable population groups as social inequalities are thought to be aggravated by events that lead to further restriction of access to oral healthcare services such as closure of dental practices during the COVID-19 pandemic [33].

Water fluoridation is another key determinant of caries prevalence at country level. Whereas inadequate fluoride intake could render people more susceptible to dental caries, [34] higher fluoride doses result in poorly mineralized porous enamel in severe cases increasing caries risk [35]. The United States Public Health Service [36] recommends optimal fluoride levels in drinking water of 0.7; however, Ahmed et al. [37], reported that fluoride concentrations in the groundwater of the Nile Valley ranged between 0.11 and 0.45 with an average of 0.24 mg/l. Conversely, high fluoride levels were found in drinking tap water in the Nile Delta region ranging between 0.92 and 3.75 with a mean of 1.9 mg/l which exceeds the recommended limits [38]. The elevated caries risk among well water drinkers, residing mainly in Deserts/frontier region, could be thus, linked to exposure to inappropriate fluoride levels and further aggravated by the extreme poverty and lack of dental services in those areas.

Our work has benefited from several strengths, primarily the large sample size and its high level of representativeness which allow statistical generalization of study findings to the target population. Besides, being a population-based study allows generating hypotheses to be tested in future research, monitoring changing trends in dental caries and assessing progress towards set national goals. It is also noteworthy that, while past caries experience provides information on the natural history of the disease and its lifetime prevalence, it generates no evidence on rates of untreated caries lesions which are more relevant for measuring disease burden and planning programs for disease control and prevention. One strength of the current study was investigating the proportion of population having untreated caries and potential risk indicators given that currently estimation of disease burden is mainly based on "presence of a disability" [39].

This study, however, has limitations. Owing to its cross-sectional design, the directionality of association between dental caries and tested risk indicators could not be determined and no inferences about causality could be made [40]. Furthermore, self-reporting of the causes of tooth loss is prone to recall bias, which could have led to under or overestimation of the participants' past caries experience [41]. Using DMFT index gives rise to further inherent limitations since it only considers cavitated teeth as decayed which further underestimates the prevalence of caries. Moreover, it gives equal weights to all index components; hence, it does not differentiate between carious and well-restored teeth. The index, however, has advantages that merit its use such as feasibility in remote areas, validity, reliability, and allowing international comparability of disease burden [42].

### Conclusions

Dental caries remains a major health issue in Egypt particularly among vulnerable groups namely women and people with a low SEP. Given the high proportion of untreated carious lesions among young Egyptian adults, preventive approaches such as regular screening and provision of interim or conservative restorative techniques are highly recommended in early years of life as proposed by the life course approach. Routine screening of different public water systems throughout the country is also warranted to adjust fluoridation levels and promote substantial caries prevention at national level.

## Data Availability

The datasets analysed for the current study can be made available from the corresponding author upon reasonable request.

## Abbreviations

CAPMAS: Central Agency for Public Mobilization and Statistics
CI: Confidence interval
CPI: Community Periodontal index
DMFT: Decayed, missed, filled teeth
DT: Decayed Teeth
MOHP: Ministry of Health and Population
OR: Odds Ratio
SEP: Socioeconomic position
WHO: World Health Organization
